# A Review of Diabetes Prediction Equations in African Descent Populations

**DOI:** 10.3389/fendo.2019.00663

**Published:** 2019-10-01

**Authors:** Regine Mugeni, Jessica Y. Aduwo, Sara M. Briker, Thomas Hormenu, Anne E. Sumner, Margrethe F. Horlyck-Romanovsky

**Affiliations:** ^1^National Institute of Diabetes and Digestive and Kidney Diseases, National Institutes of Health, Bethesda, MD, United States; ^2^National Institute of Minority Health and Health Disparities, National Institutes of Health, Bethesda, MD, United States; ^3^Brooklyn College, City University of New York, Brooklyn, NY, United States

**Keywords:** African (Black) diaspora, prediction equation, diabetes detection, diabetes risk, African descent population

## Abstract

**Background:** Predicting undiagnosed diabetes is a critical step toward addressing the diabetes epidemic in populations of African descent worldwide.

**Objective:** To review characteristics of equations developed, tested, or modified to predict diabetes in African descent populations.

**Methods:** Using PubMed, Scopus, and Embase databases, a scoping review yielded 585 research articles. After removal of duplicates (*n* = 205), 380 articles were reviewed. After title and abstract review 328 articles did not meet inclusion criteria and were excluded. Fifty-two articles were retained. However, full text review revealed that 44 of the 52 articles did not report findings by AROC or C-statistic in African descent populations. Therefore, eight articles remained.

**Results:** The 8 articles reported on a total of 15 prediction equation studies. The prediction equations were of two types. Prevalence prediction equations (*n* = 9) detected undiagnosed diabetes and were based on non-invasive variables only. Non-invasive variables included demographics, blood pressure and measures of body size. Incidence prediction equations (*n* = 6) predicted risk of developing diabetes and used either non-invasive variables or both non-invasive and invasive. Invasive variables required blood tests and included fasting glucose, high density lipoprotein-cholesterol (HDL), triglycerides (TG), and A1C. Prevalence prediction studies were conducted in the United States, Africa and Europe. Incidence prediction studies were conducted only in the United States. In all these studies, the performance of diabetes prediction equations was assessed by area under the receiver operator characteristics curve (AROC) or the C-statistic. Therefore, we evaluated the efficacy of these equations based on standard criteria, specifically discrimination by either AROC or C-statistic were defined as: Poor (0.50 – 0.69); Acceptable (0.70 – 0.79); Excellent (0.80 – 0.89); or Outstanding (0.90 – 1.00). Prediction equations based only on non-invasive variables reported to have poor to acceptable detection of diabetes with AROC or C-statistic 0.64 – 0.79. In contrast, prediction equations which were based on both non-invasive and invasive variables had excellent diabetes detection with AROC or C-statistic 0.80 – 0.82.

**Conclusion:** Equations which use a combination of non-invasive and invasive variables appear to be superior in the prediction of diabetes in African descent populations than equations that rely on non-invasive variables alone.

## Introduction

Predicting undiagnosed type 2 diabetes is a critical step toward addressing the diabetes epidemic in populations of African descent worldwide (1). Screening strategies should identify people of African descent at high risk of diabetes so that referrals for further testing and intervention can be made. Prediction equations developed in white or multi-ethnic population studies (e.g., white, African American, Asian, and Hispanic) may not perform well in African-descent populations. In fact, diabetes risk factors such as body mass index (BMI), waist circumference, fasting plasma glucose, triglycerides, high density lipoprotein (HDL), triglyceride/HDL-cholesterol-ratio, and hemoglobin A1c (A1C) have different thresholds of risk in African immigrants in the US compared to African Americans, and whites (2–6). African immigrants appear to have higher risk of diabetes at lower BMI, different waist circumference cut-off and a younger age than African Americans (2, 7–9). Furthermore, fasting glucose may be lower in African-descent than white populations and this may be due to lower hepatic fat, less hepatic insulin resistance and a lower rate of hepatic gluconeogenesis (10, 11). In addition, low normal triglyceride (TG) levels even in the presence of insulin resistance in African-descent populations may also lead to an underestimation of diabetes risk (12, 13). Furthermore, A1C as a non-fasting marker in Africans may underestimate glycemia because of micronutrient deficiencies, and genetic factors related to African ancestry such as hemoglobinopathies and G6PD deficiency (14–19).

### Types of Prediction Equations

There are two major types of diabetes prediction equations: Prevalence prediction equations and incidence prediction equations (20). Prevalence prediction equations are designed to detect undiagnosed diabetes cases in cross-sectional cohorts; whereas incidence prediction equations are designed to predict the risk of developing diabetes in the future and are based on longitudinal cohorts (20). Prediction equations may be simplified and reported as diabetes risk scores for easier screening classification (21).

Variables in the prediction equations are either non-invasive or invasive (22). Non-invasive variables include questions about medical history or physical measurements and require no blood to be drawn. Invasive variables require blood tests. Blood tests include plasma, serum or whole blood and require laboratory facilities to analyze blood samples (22).

To develop a prediction equation, both the risk factors and the outcome must be known (prevalence prediction) or become known (incidence prediction). The contribution of each risk factor is assessed statistically, most often through logistic regression or Cox proportional hazard (23). Prediction equations are evaluated by their ability to discriminate between patients who are at risk of a particular dichotomized outcome and those who are not at risk. Discrimination measures the ability of the prediction equation to assign a higher probability of the outcome (sensitivity) to those with the disease and a lower probability of the outcome (specificity) to those who do not have the disease (24).

The objectives of this study were (a) to conduct a review of current diabetes risk prediction equations and risk scores developed, validated, tested, or optimized to detect incident or prevalent diabetes in African descent populations living in Africa or the diaspora; and (b) summarize the predictive value of these diabetes prediction equations.

## Methods

In December 2018, a literature search of peer reviewed journals from PubMed, Scopus and Embase was performed. The list of search terms is available in Supplementary Material 1.

Studies which met the following inclusion criteria were included:

- Original studies published between January 2000 and December 2018 examining the development, calibration, validation or performance of one or more diabetes prediction equations predicting prevalent or incident type 2 diabetes;
- Study populations were exclusively or partially of black African descent;
- Study participants were identified as black populations in sub-Saharan Africa; or as black, African, African American, or designated as African-descent by a compound ethnic label such as Afro-Caribbean, living in the diaspora;
- Each prediction equation was constructed based on logistic regression analysis assessing the contribution of each predictor variable;
- Predictors in equations included any combination of two or more demographic, behavioral, historical, clinical, anthropometric, hematological, chemical, or biochemical variables;
- Prediction equations estimated type 2 diabetes incidence or prevalence;
- Diabetes outcome was determined by contemporary criteria at the time of the study by the American Diabetes Association or self-reported diabetes diagnosis or diabetes treatment;
- Performance of prediction equations for the detection of diabetes was assessed by area under the receiver operator characteristics curve (AROC) or C-statistic; and results were reported specifically for the African-descent population.

Studies predicting type 1 diabetes, gestational diabetes, diabetes-related complications and mortality, diabetes secondary to HIV, chemotherapy or organ transplant, or risk of chronic disease in patients with diabetes were excluded. Studies predicting diabetes based on a single criterion, genetic risk scores or machine learning were excluded. In addition, studies predicting diabetes in multiethnic populations which included African-descent populations but did not report results by race were also excluded.

## Results

**Figure 1:**
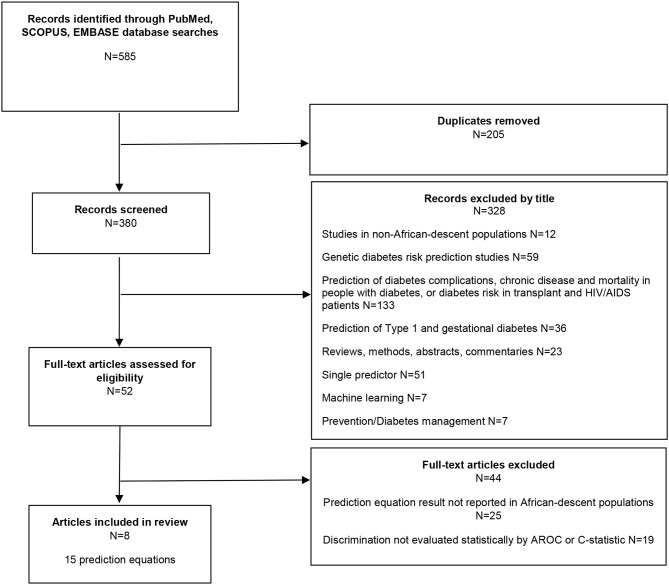
Diabetes prediction equation studies: literature review flowchart, diabetes prediction equations in African descent populations.

The literature review identified 585 research articles. After removal of duplicates, 380 articles were reviewed. A total of 52 articles were retained after title and abstract review. After full text review, eight articles which met our inclusion criteria remained (Figure 1).

The eight articles evaluated 15 individual diabetes prediction equation studies. Nine studies predicted prevalence (25–30) (Equations 1–9) (Table 1A), and six studies predicted incidence (30–32) (Equation 10–15) (Table 1B). Of these, 14 studies evaluated the performance of an existing diabetes prediction equation (Equations 1–8 and 10–15); and one study developed a new diabetes prediction equation (Equation 9). Seven equation studies were conducted in the United States (29–32), six in Africa [South Africa (26) and Botswana (28)], and two in Europe [United Kingdom (25) and the Netherlands (27)]. The fifteen equations contained 12 non-invasive variables (Table 2). Four invasive variables were used in incidence prediction equations only. They were; fasting glucose, high density lipoproteins (HDL), triglycerides (TG) and A1C. Of these fasting glucose and HDL were the most frequently used (Table 2).

**Table 1:** Prevalence and incidence diabetes prediction equation studies: study populations.

	**Equation**	**Reference**	***N***	**Age (Years)**	**Country**	**Population**
**A. Prevalence prediction equation studies**						
1	Cambridge Risk Score	Masconi et al. (26)	737	15–95	South Africa	South African Mixed-Ancestry^*^: 100%
2	Cambridge Risk Score	Spijkerman et al. (25)	803	40–75	United Kingdom	Black Caribbean: 31% South Asian: 69%
3	FINDRISC, Original	Omech et al. (28)	291	>20	Botswana	Black: 100%
4	FINDRISC, Original	Zhang et al. (29)	20,633	≥20	United States	Black: 18% Hispanic: 25% White: 53% Other: 4%
5	FINDRISC, Simplified	Masconi et al. (26)	737	15–95	South Africa	South African Mixed-Ancestry^*^: 100%
6	Kuwaiti	Masconi et al. (26)	737	15–95	South Africa	South African Mixed-Ancestry^*^: 100%
7	Omani	Masconi et al. (26)	737	15–95	South Africa	South African Mixed-Ancestry^*^: 100%
8	Rotterdam Predictive Model	Masconi et al. (26)	737	15–95	South Africa	South African Mixed-Ancestry^*^: 100%
9	SUNSET Risk Score	Bindraban et al. (27)	1,415	35–60	The Netherlands	African Surinamese: 42% Hindustani Surinamese: 24% Ethnic Dutch: 34%
**B. Incidence prediction equation studies**						
10	ARIC	Lacy et al. (31)	2,456	18–30	United States	Black: 15% White: 85%
11	ARIC	Mann et al. (32)	5,329	45–84	United States	Black 25% Hispanic 21% Chinese 12% White 43%
12	ARIC+A1C	Lacy et al. (31)	999	18–30	United States	Black: 100%
13	FINDRISC, Modified	Kulkarni et al. (30)	9,754	45–64	United States	Black:18% White: 82%
14	Framingham Offspring Study	Mann et al. (32)	5,329	45–84	United States	Black 25% Hispanic 21% Chinese 12% White 43%
15	San Antonio Heart Study	Mann et al. (32)	5,329	45–84	United States	Black 25% Hispanic 21% Chinese 12% White 43%

* *Mixed-ancestry population in South Africa: Ancestral components: Khoesan (32–43%) [Black], Bantu-speaking Africans (20–36%) [Black], European (21–28%), and a smaller Asian contribution (9–11%) (33)*.

**Of African Surinamese, 99.2% were born in Surinam and 99.5% had two parents born in Surinam. 79.3% of the African Surinamese had two parents who were of African origin. (27).

**Table 2:** Diabetes prediction equation studies: variables included in 15 diabetes equations.

	**Number of equations which include variable**
**NON-INVASIVE VARIABLES**	
**Subjective/medical history**	
Age	14
Family history of diabetes	12
Prescription medication, hypertension	9
Sex	4
Race/Ethnicity	4
Hypertension	3
History of high blood glucose	2
Physical activity	2
Prescription medications, steroids	2
Smoking	2
Diet (Fruit and vegetable consumption)	2
Family history of cardiovascular disease	1
**Clinical/Measured**	
BMI	11
Waist circumference	10
Blood pressure, Systolic	4
Height	3
Heart rate	1
**INVASIVE VARIABLES**	
**Plasma or Serum**	
Fasting glucose	5
High density lipoprotein	5
Triglycerides	4
**Whole Blood**	
Hemoglobin A1C	1

Study populations varied widely in size ranging from 291 to 20,633 participants (Table 1). African descent populations made up between 15 and 100% of the study cohorts. All prediction studies reported performance in African descent population groups by AROC or C-statistic (Table 3).

**Table 3:** Diabetes prediction equation studies: variables, results, and main findings.

**Equation number**	**Equation**	**Reference**	**Diabetes definition**	**Biomarkers/variables**	**Results in African descent population ONLY**	**Diabetes discrimination in African descent population ONLY**
**A. Prevalence prediction equation studies**						
1	Cambridge Risk Score	Masconi et al. (26)	Fasting glucose ≥126 mg/dL or 2 h OGTT ≥200 mg/dL	Sex, HTN Rx, steroid Rx, age, BMI, family Hx of DM, smoking	C-statistic: 0.67 (Total Pop) C-statistic: 0.67 (men) C-statistic: 0.67 (women)	Poor
2	Cambridge Risk Score	Spijkerman et al. (25)	Fasting glucose ≥126 mg/dL or A1C ≥6.5%^#^	Sex, HTN Rx, steroid Rx, age, BMI, family Hx of DM, smoking	AROC: 0.67	Poor
3	FINDRISC, Original	Omech et al. (28)	A1C ≥6.5%	Age, BMI, WC, HTN Rx, family Hx DM, daily fruit/berries/vegetables, physical activity	AROC: 0.63	Poor
4	FINDRISC, Original	Zhang et al. (29)	Fasting glucose ≥126 mg/dL or 2h OGTT ≥200 mg/dL or A1C ≥6.5% or Self-reported diagnosis of diabetes	Age, BMI, WC, HTN Rx, family Hx DM, daily fruit/berries/vegetables, physical activity	AROC: 0.76	Acceptable
5	FINDRISC, Simplified	Masconi et al. (26)	Fasting glucose ≥126 mg/dL or 2 h OGTT ≥200 mg/dL	Age, BMI, WC, HTN Rx, Hx high blood glucose	C-statistic: 0.67 (Total Pop) C-statistic: 0.70 (men) C-statistic: 0.66 (women)	Poor
6	Kuwaiti	Masconi et al. (26)	Fasting glucose ≥126 mg/dL or 2h OGTT ≥200 mg/dL	Family Hx of DM, HTN Rx, age, WC	C-statistic: 0.68 (Total Pop) C-statistic: 0.70 (men) C-statistic: 0.67 (women)	Poor
7	Omani	Masconi et al. (26)	Fasting glucose ≥126 mg/dL or 2 h OGTT ≥200 mg/dL	Age, WC, BMI, family Hx of DM, hypertension	C-statistic: 0.66 (Total Pop) C-statistic: 0.62 (men) C-statistic: 0.66 (women)	Poor
8	Rotterdam Predictive Model	Masconi et al. (26)	Fasting glucose ≥126 mg/dL or 2 h OGTT ≥200 mg/dL	Age, sex, HTN Rx, BMI	C-statistic: 0.64 (Total Pop) C-statistic: 0.62 (men) C-statistic: 0.66 (women)	Poor
9	SUNSET Risk Score	Bindraban et al. (27)	Fasting glucose ≥126 mg/dL and/or Self-reported diagnosis of diabetes	Age, BMI, WC, resting heart rate, family Hx of DM, hypertension or HTN Rx, family Hx of CVD, ethnicity(race)	AROC 0.79	Acceptable
**B. Incidence prediction equation studies**						
10	ARIC	Lacy et al. (31)	ADA 2004: Fasting glucose ≥126 mg/dL or 2 h OGTT ≥200 mg/dL ADA 2010: Fasting glucose ≥126 mg/dL or 2 h OGTT ≥200 mg/dL or A1C ≥6.5%	Age, race, parent Hx of DM, fasting glucose, SBP, WC, HGT, HDL, TG.	AROC: 0.80 (ADA 2004) AROC: 0.80 (ADA 2010 Re-estimated)	Excellent
11	ARIC	Mann et al. (32)	Fasting glucose ≥126 mg/dL or Self-reported hypoglycemic Rx or insulin	Age, race, parent Hx of DM, fasting glucose, SBP, WC, HGT, HDL, TG	C-statistic: 0.81	Excellent
12	ARIC+A1C	Lacy et al. (31)	Fasting glucose ≥126 mg/dL or 2 h OGTT ≥200 mg/dL or A1C ≥6.5%	Age, parent Hx of DM, fasting glucose, SBP, WC, HGT, HDL, TG, A1C	AROC: 0.82	Excellent
13	FINDRISC, Modified	Kulkarni et al. (30)	Fasting glucose ≥126 mg/dL or Self-reported Dx of diabetes by a physician or Self-reported hypoglycemic Rx	Age, BMI, WC, HTN Rx, Hx hyperglycemia	AROC: 0.70 (men) AROC: 0.71 (women)	Acceptable
14	Framingham Offspring Study	Mann et al. (32)	Fasting glucose ≥126 mg/dL or Self-reported hypoglycemic Rx or insulin	Fasting glucose, BMI, HDL, TG, hypertension, family Hx of DM	C-statistic: 0.80	Excellent
15	San Antonio Heart Study	Mann et al. (32)	Fasting glucose ≥126 mg/dL or Self-reported hypoglycemic Rx or insulin	Age, sex, race, fasting glucose, SBP, HDL, BMI, family Hx of DM.	C-statistic: 0.80	Excellent

*T2DM, Type 2 diabetes mellitus; DM, diabetes mellitus; Hx, history; Rx, prescription; HGT, height; FPG, fasting plasma glucose; FINDRISC, Finnish Diabetes Risk Score; HDL, high density lipoprotein; SBP, systolic blood pressure; WC, waist circumference; TG, triglycerides; OGTT, oral glucose tolerance test; AROC, area under the receiver operator characteristic curve; CVD, cardiovascular disease; HTN Rx, Anti-hypertension medication; BMI, Body mass index; ARIC, Atherosclerosis Risk in Communities*.

# *Although the ADA diagnostic criteria for diabetes did not include A1C at the time of this study, Spijkerman et al. who based their study design on survey data cited the value of using A1C for diabetes diagnosis in the absence of fasting plasma glucose values*.

### Diabetes Outcome Definitions

Diabetes was defined as either prevalent or incident. Diabetes diagnosis was based on one or more American Diabetes Association (ADA) criteria at the time studies were conducted. For studies published between 2004 and 2010, diabetes was diagnosed by fasting glucose ≥126 mg/dL or 2-h oral glucose tolerance test (OGTT) ≥200 mg/dL. However, Spijkerman et al. (Equation 2) also used A1C ≥6.5% to diagnose diabetes in the absence of fasting glucose or 2-h glucose from the OGTT, before A1C was added to standard ADA criteria (25, 34). For two studies conducted after 2010 (Equations 3, 10, and 12) diabetes diagnosis criteria included A1C ≥6.5% in addition to fasting and 2-h OGTT glucose criteria. Furthermore, for six equations (Equations 4, 9, 11, 13, 14, and 15), diabetes outcome was also defined as self-reported diagnosis of diabetes (e.g., Having been told they had diabetes by a physician or other medical professional); medical record documentation of a diabetes diagnosis; or self-reported initiation of oral hypoglycemic or insulin treatment (Tables 1, 3).

### Development and Measures of Performance of Diabetes Prediction Equations

Diabetes risk variables significantly associated with the outcome were included in a multivariate logistic regression model which estimated the β-coefficient for each of these variables. The risk calculation was then based on the contribution of each variable included in the model. The probability of diabetes was calculated based on the sum of the prediction equation (27, 35). All studies reviewed here, except one (Equation 9), evaluated, or validated the performance of prediction equations which had originally been developed in other cohorts. Bindraban et al. developed a new equation (Equation 9) in the study population in which it was reported. See Table 4 for the full equation for each study.

**Table 4:** Diabetes prediction equations.

**A. Prevalence prediction equation studies**	
**1**	**Cambridge Risk Score, Masconi et al**. **(****26****)**
	Probability (DM) = exp(X)/(1 + exp(X)); where X = −6.322 – [0.879 (if female, else 0)] + [1.222 (if HTN Rx, else 0)] + [2.191 (if steroids Rx, else 0)] + [0.063* age (y)] + [0.699 (if 25 ≤ BMI ≤ 27.49 kg/m^2^ else 0)] + [1.970 (if 27.5 ≤ BMI ≤ 29.99 kg/m^2^, else 0)] + [2.518 (if BMI ≥ 30 kg/m^2^, else 0)] + [0.728 (if parent or sibling Hx DM, else 0)] + [0.753 (if parent and sibling Hx DM, else 0)] – [0.218 (if ex-smoker, else 0)] + [0.855 (if current smoker, else 0)]
	The Cambridge Risk Score was originally derived in a United Kingdom population (*N* = 1,077^+^) (34)
**2**	**Cambridge Risk Score, Spijkerman et al**. **(****25****)**
	Probability (DM) = 1/(1+exp-(X)), where X = −6.322 – [0.879 (if female, else 0)] + [1.222 (if HTN Rx, else 0)] + [2.191 (if steroids Rx, else 0)] + [0.063* age (y)] + [0.699 (if 25 ≤ BMI ≤ 27.49 kg/m^2^, else 0)] + [1.970 (if 27.5 ≤ BMI ≤ 29.99 kg/m^2^, else 0)] + [2.518 (if BMI ≥ 30 kg/m^2^, else 0)] + [0.728 (if parent or sibling Hx DM, else 0)] + [0.753 (if parent and sibling Hx DM, else 0)] – [0.218 (if ex-smoker, else 0)] + [0.855 (if current smoker, else 0)]
	The Cambridge Risk Score was originally derived in a United Kingdom population (*N* = 1,077^+^) (34)
**3**	**FINDRISC, Original, Omech et al**. **(****28****)**
	Probability (DM) = 1/(1+exp-(X)), where X = [0 (if age <45y)] + [2 (if 45 ≤ age ≤ 54y, else 0)] + [3 (if 55 ≤ age ≤ 64y, else 0)]+[4 (if age > 64y)]+ [0 (if BMI <25 kg/m^2^)] + [1 (if 25 ≤ BMI ≤ 30 kg/m^2^, else 0)] + [3 (if BMI >30 kg/m^2^), else 0] +[0 (if men: WC <94 cm)]+ [3 (if men: 94 ≤ WC ≤ 102 cm, else 0)] + [0 (if women: WC <80 cm)]+[3 (if women: 80 ≤ WC ≤ 88 cm, else 0)] + [4 (if men: 102 cm < WC, else 0)] + [4 (if women: 88 cm < WC, else 0)] + [2 (if HTN Rx, else 0)] + [5 (if family Hx of high blood glucose, else 0)] + [2 (if daily physical activity >30 min, else 0)] + [1 (if daily consumption of vegetables/fruits/berries, else 0)] + [5 (If family Hx of DM in parent, brother, sister, child, else 0)] + [3 (if family history of DM in grandparent, aunt, uncle or first cousin, else 0)] ^#^
	The FINDRISC was originally derived in a Finnish population (*N* = 4,746^+^) (35)
**4**	**FINDRISC, Original, Zhang et al**. **(****29****)**
	Probability (DM) = 1/(1+exp-(X)), where X = [0 (if age <45y)] + [2 (if 45 ≤ age ≤ 54y, else 0)] + [3 (if 55 ≤ age ≤ 64y, else 0)]+[4 (if age > 64y)]+ [0 (if BMI <25 kg/m^2^)] + [1 (if 25 ≤ BMI ≤ 30 kg/m^2^, else 0)] + [3 (if BMI >30 kg/m^2^), else 0] +[0 (if men: WC <94 cm)]+ [3 (if men: 94 ≤ WC ≤ 102 cm, else 0)] + [0 (if women: WC <80 cm)]+[3 (if women: 80 ≤ WC ≤ 88 cm, else 0)] + [4 (if men: 102 cm < WC, else 0)] + [4 (if women: 88 cm < WC, else 0)] + [2 (if HTN Rx, else 0)] + [5 (if family Hx of high blood glucose, else 0)] + [2 (if daily physical activity >30 min, else 0)] + [1 (if daily consumption of vegetables/fruits/berries, else 0)] + [5 (If family Hx of DM in parent, brother, sister, child, else 0)] + [3 (if family history of DM in grandparent, aunt, uncle or first cousin, else 0)] ^#^
	The FINDRISC was originally derived in a Finnish population (*N* = 4,746^+^) (35)
**5**	**FINDRISC, Simplified, Masconi et al**. **(****26****)**
	Probability (DM) = exp(X)/(1 + exp(X)); where X = −5.514 + [0.628 (if 45 ≤ age ≤ 54y, else 0)] + [0.892 (if 55 ≤ age ≤ 64y, else 0)] + [0.165 (if 25 ≤ BMI <30 kg/m^2^, else 0)] + [1.096 (if BMI > 30 kg/m^2^, else 0)] + [0.857 (if men: 94 ≤ WC <102 cm) or (if women: 80 ≤ WC <88 cm)] + [1.350 (if men: WC ≥ 102 cm) or (if women: WC ≥ 88 cm)] + [0.711 (if HTN Rx, else 0)] + [2.139 (if Hx of hyperglycemia, else 0)]
	The FINDRISC was originally derived in a Finnish population (*N* = 4,746^+^) (35)
**6**	**Kuwaiti, Masconi et al**. **(****26****)**
	Probability (DM) = exp(X)/(1 + exp(X)), where X = −5.018 + [0.979 (If sibling Hx DM, else 0)] + [0.978 (if anti-HTN Rx, else 0)] + [1.315 (if Age ≥ 35y)] + [1.930 (if WC≥100 cm, else 0)]
	The Kuwaiti Diabetes prediction equation was originally derived in a Kuwaiti population (*N* = 562^+^) (36)
**7**	**Omani, Masconi et al**. **(****26****)**
	Probability (DM) = exp(X)/(1 + exp(X)); where X = −4.7 + [1.8 (if 40 ≤ age ≤ 59y)] + [2.3 (if age ≥ 60y)] + [0.38 (if Men WC ≥ 94 cm; or Women WC ≥ 80 cm)] + [0.54 (if 25 ≤ BMI <30 kg/m^2^, else 0)] + [0.69 (if BMI ≥ 30 kg/m^2^, else 0)] + 1.9 (if parent or sibling Hx DM, else 0]) + [0.73 (if SBP≥140 mm Hg and/or DBP ≥90 mm Hg)]
	The Omani diabetes prediction equation was originally derived in an Omani population (*N* = 4,881) (37)^+^
**8**	**Rotterdam Predictive Model, Masconi et al**. **(****26****)**
	Probability (DM) = exp(X)/(1 + exp(X)); where X = −3.02 + [0.19 (per 5-year increment from 55 to >75 years, else 0)] + [0.46 (if male, else 0)] + [0.42 (if anti-HTN Rx, else 0) + 0.51 (if BMI ≥ 30 kg/m^2^, else 0)
	The Rotterdam Predictive Model was originally derived in a Dutch population (*N* = 1,016^+^) (38)
**9**	**SUNSET Risk Score, Bindraban et al**. **(****27****)**
	Probability (DM) = 1/(1+ exp(-X)), where X = −1.638+ [0.293 (If age ≥ 45y)v + [0.317 (if BMI >25 kg/m^2^ or BMI>23 kg/m^2^ for Hindustani Surinamese, else 0)] + [0.411 (if women: WC >80 cm; if black or white European men WC >94 cm; if Hindustani Surinamese men WC >90 cm, else 0)] + [0.433 (if resting heart rate ≥ 90 bpm, else 0] + [0.497 (if family Hx DM, else 0)] + [0.433 (if BP > 140/90 mmHg and/or anti-HTN Rx, else 0)] + [0.555 (if Hx CVD, else 0)] + [0 (if ethnic Dutch)] + [−0.084 (if African Surinamese)] + [0.547 (if Hindustani Surinamese)]
	The SUNSET Risk Score was originally derived in a multiethnic Dutch population (*N* = 1,415, 42% African Surinamese, 24% Hindustani Surinamese and 34% Ethnic Dutch) (27)
**B. Incidence prediction equation studies**	
**10**	**ARIC, Lacy et al**. **(****31****)**
	Probability (DM) = [[exp(X)/(1+exp(X))]/9]*5, where X * =* −9.9808 + [0.0173*age (y)] + [0.4433 (if African American, else 0)] + [0.4981(if parent Hx DM, else 0)]+ [0.0880*FPG (mg/dL)] + [0.0111*SBP (mmHg)] + [0.0273*WC (cm)] – [0.0326*Hgt (cm)] – [0.0122*HDL (mg/dL)] + [0.00271*TG (mg/dL)].
	The ARIC diabetes prediction equation was originally derived in a multiethnic United States population (*N* = 7,915, 85% white) (39)
**11**	**ARIC, Mann et al**. **(****32****)**
	Probability (DM) = [[exp(X)/(1+exp(X))]/9]*4.75, where X = 12.911 – [0.305 *age (y)] + [0.181 (if African American, else 0)] + [0.578 (if parent Hx of DM, else 0)] + [0.119*FPG (mg/dL)] + [0.006*SBP (mm Hg)] + [0.028 *WC (cm)]+ [0.015*Hgt (cm)] -[0.009 x HDL (mg/dL)] + [0.001*TG (mg/dL)]
	The ARIC diabetes prediction equation was originally derived in a multiethnic United States population (*N* = 7,915, 85% white) (39)
**12**	**ARIC+A1C, Lacy et al**. **(****31****)**
	Probability (DM) = [[exp(X)/(1+exp(X))]/9]*5, where X = −19.9786 + [0.0234*age (y)] + [0.2713*parent Hx DM (1/0)] + [0.0587*FPG (mg/dL)] + [0.0184*SBP (mmHg)] + [0.0242*WC (cm)] + [−0.0124*HGT (cm)] + [-0.0144*HDL (mg/dl)] + [−0.00044*TG (mg/dL)] + [1.6237*A1C (%)]
	The ARIC+A1C diabetes prediction equation was originally derived in a multiethnic United States population (*N* = 7,915, 85% white) (39) and updated in another multiethnic United States population (*N* = 2,456, 41% black, 59% white) (31)
**13**	**FINDRISC, Modified, Kulkarni et al**. **(****30****)**
	Probability (DM) = 1/(1+exp-(X)), where X = + [2 (if 45 ≤ age ≤ 54 years, else 0)] + [3 (if 55 ≤ age ≤ 64 years, else 0)]+ [0 (if BMI <25 kg/m^2^)] + [1 (if 25 ≤ BMI ≤ 30 kg/m^2^, else 0)] + [3 (if BMI >30 kg/m^2^), else 0] +[0 (if men: WC <94 cm)]+ [3 (if men: 94 ≤ WC ≤ 102 cm, else 0)] + [0 (if women: WC <80 cm)]+[3 (if women: 80 ≤ WC ≤ 88 cm, else 0)] + [4 (if men: 102 cm < WC, else 0)] + [4 (if women: 88 cm < WC, else 0)] + [2 (if HTN Rx, else 0)] + [5 (if family Hx of high blood glucose, else 0)]^@^, ^&^
	The FINDRISC was originally derived in a Finnish population (*N* = 4,746^+^) (35)
**14**	**Framingham Offspring Study Diabetes Prediction Equation, Mann et al**. **(****32****)**
	Probability (DM) = [[exp(X)/(1+exp(X))]/8]*4.75, where X = 4.281 + [2.26 (if impaired FPG, else 0)] + [0.157 (if 25 < BMI ≤ 30 kg/m^2^, else 0)] + [0.189 (if 30 kg/m^2^ < BMI, else 0)] + [0.063 (If low HDL, else 0)] + [0.082 (if high TG, else 0)] + [0.157 (if elevated BP, else 0)] + [0.211(if parent Hx of DM, else 0)]
	The Framingham Offspring Study Diabetes Prediction Equation was originally derived in a United States population (*N* = 3,140, 99% non-Hispanic white) (40)
**15**	**San Antonio Heart Study Diabetes Prediction Equation, Mann et al**. **(****32****)**
	Probability (DM) = [[exp(X)/(1+exp(X))]/7.5]*4.75, where X = 14.836 – [0.239 * age (y)] + [0.367 (if female, else 0)] – [0.129 (if Mexican, else 0)] + [0.122 *FPG (mg/dL)] + [0.006* SBP (mm Hg)] + [0.016* HDL (mg/dL)] + [0.034 *BMI (kg/m^2^)] + [0.567 (if family Hx DM, else 0)]
	The San Antonio Heart Study Diabetes Prediction Equation was originally derived in a multiethnic United States population (*N* = 2,903, 62% Hispanic, 38% non-Hispanic white) (41)

+ *No ethnicity specified*.

# *Equation was assumed to be the same as the FINDRISC Original from the cross-sectional validation study by Saaristo et al. (42)*.

@ Equation was assumed to be the same as the original FINDRISC Concise model from the original study by Lindstrom et al. (35).

& The study population included only participants aged 45–64-year-old. The FINDRISC adds 0 points for ages <45 years and 4 points for ages >64 (35).

*T2DM, Type 2 diabetes mellitus; DM, diabetes mellitus; Hx, history; Rx, prescription; HGT, height; FPG, fasting plasma glucose; FINDRISC, Finnish Diabetes Risk Score; HDL, high density lipoprotein; SBP, systolic blood pressure; WC, waist circumference; TG, triglycerides; OGTT, oral glucose tolerance test; AROC, area under the receiver operator characteristic curve; CVD, cardiovascular disease; HTN Rx, Anti-hypertension medication; BMI, Body mass index; ARIC, Atherosclerosis Risk in Communities*.

Performance of prediction equations was evaluated by several statistical methods. In this review we focused on studies summarizing the ability to predict diabetes by either AROC or C-statistic.

AROC assesses how well each equation distinguishes or discriminates between patients who have diabetes and those who do not. A score of 0.50 indicates no discrimination; 0.50<AROC<0.70 poor discrimination; 0.70≤AROC<0.80 acceptable discrimination; 0.80≤AROC<0.90 excellent discrimination; 0.90≤AROC outstanding discrimination; and a score of 1.00 perfect discrimination (45).

The C-statistic estimates a higher risk for the person who has (a prevalent case) or develops diabetes (an incident case) compared to the risk assigned to the person who does not have or does not develop diabetes. C-statistic measures the concordance between predicted and observed outcomes and range from 0.50 (random concordance) to 1.00 (perfect concordance). The C-statistic is seen as equal to AROC (46).

Performance of prediction equations was assessed by AROC for seven studies and by C-statistic for eight studies.

### Prevalence Prediction Studies

Five articles included nine prevalence prediction equation studies which were conducted in South Africa, United Kingdom, Botswana, United States and the Netherlands. Table 1A lists study populations for each of these studies.

The nine prevalence prediction equations included only non-invasive variables: age, sex, family history, health behavior, medical history, anthropometric and clinical risk factors. Prevalence prediction equations contained three to nine variables (Table 3A). Five prevalence prediction studies reported results for the overall study population as well as by sex (Equations 1, 5–8).

#### Equation 1 and 2: The Cambridge Risk Score

The Cambridge risk score was tested in South Africa (26) (Equation 1) and United Kingdom (25) (Equation 2).

For equation 1, the South African cross-sectional study had 737 participants (100% black mixed-ancestry, mean age 51.2 years) enrolled in the Cape Town Bellville-South cohort (26). The mixed-ancestry population in Bellville-South, South Africa is primarily of black African ancestry. Ancestral components include: Khoesan (32–43%) [Black], Bantu-speaking Africans (20–36%) [Black], European (21–28%), and Asian (9–11%) (33). Diabetes was defined as fasting glucose ≥126 mg/dL or 2 h OGTT ≥200 mg/dL.

For equation 2, the United Kingdom cross-sectional cohort (25) had 803 multiethnic participants (Black Caribbean 31%, South Asian 69%, ages 40–75) who were enrolled in the 1999 Health Survey for England. Diabetes was defined as fasting glucose ≥126 mg/dL or A1C ≥6.5%.

The Cambridge Risk Score had seven non-invasive variables: Age, sex, hypertension medication, family history of diabetes, steroid medication, BMI, and smoking status.

The Cambridge Risk Score had “poor” discrimination in both the South Africa (Equation 1) (Total African descent population: C-statistic 0.67; Men 0.67; Women 0.67) and United Kingdom study cohorts (Equation 2) (Total African descent population: AROC 0.67).

#### FINDRISC

The following three studies evaluated the FINDRISC equation in Botswana, the United States, and South Africa (Equations 3, 4, and 5). This equation was originally developed in a Finnish population to predict prevalence and incidence (37), but in the following three studies (Equations 3–5) it was used to predict prevalence.

#### Equations 3 and 4: FINDRISC (Finnish Diabetes Risk Score), Original

For equations 3 and 4, the original FINDRISC was evaluated in two cross-sectional cohorts in Botswana (28) and the United States (29).

For Equation 3, Omech et al. (28) tested the original FINDRISC (Equation 3) in Botswana. The cross-sectional cohort had 291 general medical outpatients (Assumed race 100% black, age ≥20 years, mean age 50.1). Diabetes was defined as A1C ≥6.5%.

For equation 4, Zhang et al. (29) tested the FINDRISC in the United States among 20,633 adult participants (18% black, 25% Hispanic, 53% white, 4% other; age ≥20 years, mean age 47.8) enrolled in the 1999–2010 National Health and Nutrition Examination Survey (NHANES). Diabetes was defined as fasting glucose ≥126 mg/dL, or 2 h OGTT ≥200 mg/dL, or A1C ≥6.5%.

The original FINDRISC had eight non-invasive variables: Age, BMI, waist circumference, hypertension medication, history of high blood glucose, family history of diabetes, daily fruit and vegetable consumption, and physical activity.

The original FINDRISC had had “poor” discrimination in Botswana (Equation 3) cohort (Total African descent population: AROC 0.63) and “acceptable” discrimination of diabetes in the United States (Equation 4) (Total African descent population: AROC 0.76).

#### Equation 5: FINDRISC, Simplified

For equation 5, the simplified FINDRISC was evaluated in South Africa. Masconi et al. (26) examined the simplified FINDRISC in the Cape Town Bellville-South cohort as described above.

The simplified FINDRISC included five non-invasive variables: Age, BMI, waist circumference, hypertension medication, history of high blood glucose.

The simplified FINDRISC had “poor” discrimination in the South African cohort (Equation 5) (Total African descent population: C-statistic 0.67; Men 0.70; Women 0.66).

#### Equation 6: Kuwaiti Diabetes Score

In South Africa, Masconi et al. also examined the Kuwaiti diabetes score in the Cape Town Bellville-South cohort as described above (26). The equation had four variables: Age, hypertension medication, family history, and waist circumference. The Kuwaiti equation had “poor” discrimination of diabetes in the South Africa cohort (Total African descent population: C-statistic 0.67; Men 0.70; Women 0.67).

#### Equation 7: Omani Diabetes Score

In South Africa, Masconi et al. also examined the Omani diabetes score in the Cape Town Bellville-South cohort as described above (26). The equation had five variables: Age, waist circumference, BMI, family history of diabetes, and current hypertension. The Omani score had “poor” discrimination in the South African cohort (Total African descent population: C-statistic 0.66; Men 0.62; Women 0.66).

#### Equation 8: Rotterdam Predictive Model

In South Africa, Masconi et al. also examined the Rotterdam predictive model in the Cape Town Bellville-South cohort as described above (26). The equation had four variables: Age, sex, BMI, and hypertension treatment. The Rotterdam predictive model had “poor” discrimination in the South African cohort (Total African descent population: C-statistic 0.64; Men 0.62; Women 0.66).

#### Equation 9: SUNSET Diabetes Risk Score

In the Netherlands, the SUNSET diabetes risk score (27) was a new equation derived in a cross-sectional cohort study of 1,415 participants [593 or 41% African Surinamese [Black], 336 or 23% Hindustani Surinamese [Asian], 486 or 34% ethnic Dutch (35)] ages 35–60, living in Amsterdam. Of the African Surinamese population, 99.2% were born in Surinam, 99.5% had two parents born in Surinam, and 79.3% had two parents who were of African origin. Diabetes diagnosis was defined as fasting glucose ≥126 mg/dL, or self-reported diabetes diagnosis by a physician.

The equation included eight non-invasive variables: Age, BMI, waist circumference, resting heart rate, family history of diabetes, hypertension, history of cardiovascular disease and race/ethnicity.

The SUNSET diabetes risk score had “acceptable” discrimination of diabetes (Total African descent population: AROC 0.79).

#### Performance of Prediction Equations in Prevalence Studies

Overall, seven of the nine prevalence prediction equations based on non-invasive criteria had “poor” discrimination (Equations 1, 2, 3, 5, 6, 7, and 8). Two equations had “acceptable” discrimination of diabetes (Equations 4 and 9) in study populations of African descent. AROC or C-statistic results were between 0.62 and 0.79. Six of the nine studies reported equation discrimination in both men and women. By sex, two prediction equations (Equations 5 and 6) had differential discrimination for men and women. The simplified FINDRISC and Kuwaiti equations evaluated by Masconi et al. in South Africa had “acceptable” discrimination in men, but “poor” discrimination in women. See Table 3A for diabetes prediction equation performance.

### Incidence Prediction Studies

Five incidence prediction studies employed both non-invasive and invasive variables and contained between six and nine variables. One prediction study, the modified FINDRISC (Equation 13), included only non-invasive variables (30). See Tables 3, 4 for details for each prediction equation. One incidence prediction study reported discrimination results by sex only and five studies reported results for the total African-descent population only. All incidence prediction studies were conducted in the United States.

#### Equation 10 and 11: ARIC Diabetes Prediction Equation

In the United States, Lacy et al. (31) examined the performance of the Atherosclerosis Risk in Communities (ARIC) Diabetes Prediction Equation (Equation 10), among 2,456 participants (15% African American, 85% white) ages 45–84 enrolled in the Coronary Artery Development Study in Young Adults (CARDIA) cohort study, free of diabetes at baseline. Participants were enrolled and living in four cities in the United States and followed for 5 years. Lacy et al. also evaluated the difference between the 2004 ADA diabetes criteria (diabetes diagnosis was defined as fasting glucose ≥126 mg/dL or 2 h OGTT ≥200 mg/dL); and 2010 ADA diabetes criteria (diabetes diagnosis was defined as fasting glucose ≥126 mg/dL, or 2 h OGTT ≥200 mg/dL, or A1C ≥6.5%).

In the United States, Mann et al. (32) examined discrimination by the ARIC diabetes prediction equation (Equation 11) in the multiracial cohort included 5,329 participants (25% African American, 43% white, 21% Hispanic, 12% Chinese American) in the Multi-Ethnic Study of Atherosclerosis (MESA) 2000-2002 longitudinal cohort study. Participants were free of diabetes at baseline. Ages ranged from 45 to 84. Mean age was 61.6 years where 59% of the population was <65 years of age. Participants were followed for a median of 4.75 years. Diabetes diagnosis was defined as fasting glucose ≥126 mg/dL, or self-reported hypoglycemic medication or insulin treatment.

The ARIC diabetes prediction equation had six non-invasive variables: Age, race, parent history of diabetes, systolic blood pressure, waist circumference and height; and three invasive variables: Fasting plasma glucose, HDL and triglycerides.

The ARIC equation had “excellent” discrimination in both the CARDIA (Total African descent population: AROC 0.80) and MESA (Total African descent population: C-statistic 0.81) cohorts. Lacy et al. found no difference in ARIC prediction equation performance based on the 2004 and 2010 ADA outcome definition.

#### Equation 12: ARIC+A1C Diabetes Prediction Equation

In the United States, Lacy et al. (31) added A1C to the ARIC diabetes prediction equation evaluated above and re-estimated it in a sub-sample of 999 African Americans enrolled in the CARDIA study as described above. Participants were free of diabetes at baseline and followed for 5 years. The A1C-updated ARIC diabetes prediction equation included five non-invasive variables: age, parent history of diabetes, systolic blood pressure, waist circumference and height; and four invasive variables: fasting plasma glucose, HDL, triglycerides, and A1C. The updated equation had “excellent” prediction of diabetes (Total African descent population: AROC 0.82) among African Americans.

#### Equation 13: FINDRISC, Modified

In the United States, Kulkarni et al. (30) examined the performance of the modified FINDRISC diabetes risk score, in 9,754 participants enrolled in the ARIC study cohort. Participants were (48.9% male; 18% black, 82% white; age range 45–64) free of diabetes at baseline and followed for 9 years. Diabetes was defined by fasting glucose ≥126 mg/dL or self-reported diabetes diagnosis by a physician or self-reported hypoglycemic medication. The modified FINDRISC diabetes risk score included five non-invasive variables: Age, BMI, waist circumference, hypertension medication, family history of diabetes. This prediction equation was originally developed to predict both prevalence and incidence of diabetes. In this study, Kulkarni et al. used it to predict incident cases of diabetes. The modified FINDRISC risk score had “acceptable” discrimination of diabetes in both men (AROC 0.70) and women (AROC 0.71).

#### Equation 14: Framingham Offspring Study Diabetes Prediction Equation

In the United States, the performance of the Framingham Offspring Study diabetes prediction equation was examined in the MESA 2000–2002 study cohort as described above (32).

The Framingham Offspring Study equation had three non-invasive variables: BMI, blood pressure and family history of diabetes; and three invasive variables: Fasting plasma glucose, HDL and triglycerides.

The Framingham Offspring Study equation had “excellent” discrimination of diabetes (Total African descent population: C-statistic 0.80).

#### Equation 15: San Antonio Heart Study Diabetes Prediction Equation

In the United States, the San Antonio Heart Study diabetes prediction equation (Equation 15) was examined in the MESA (32) study cohort as described above.

The equation had six non-invasive variables: Age, sex, race, systolic blood pressure, BMI, family history of diabetes; and two invasive variables: Fasting plasma glucose and HDL.

The San Antonio Heart Study diabetes equation had “excellent” prediction of diabetes (Total African descent population: C-statistic 0.80).

### Performance of Prediction Equations in Incidence Studies

Incidence prediction equations which relied on both non-invasive and invasive variables (Equations 10, 11, 12, 14, and 15) had excellent discrimination in study populations of African descent. AROC or C-statistic results were similar and between 0.80 and 0.82. In contrast, the modified FINDRISC score (Equation 13) had only “acceptable” discrimination (Total African descent population: AROC 0.70 in men, and 0.71 in women) of diabetes incidence in African-descent populations (Table 3).

## Discussion

This review identified eight diabetes prediction equation publications which assessed a total of fifteen equation studies in populations of African descent. Prediction equations were tested in African Americans in the US, Africans in Botswana, mixed-ancestry South Africans, Afro-Caribbeans in the United Kingdom and Afro-Surinamese in the Netherlands with varied results.

Prediction equations relying only on non-invasive variables had “poor” to “acceptable” detection of diabetes. In contrast, equations using both non-invasive and invasive variables had “excellent” discrimination of diabetes.

Prevalence and incidence prediction equations were originally developed in predominantly white (36, 37, 40, 42) and multi-ethnic (41, 43) populations in the United States and Europe, or Arabic (38, 39) cohorts in the Middle East. None of the prediction equations were derived in Sub-Saharan Africa. Furthermore, only equations based on non-invasive variables were tested in sub-Saharan Africa. Therefore, it is unknown how equations based on invasive risk criteria perform in sub-Saharan Africa.

Diabetes prediction equations need to be specific because African descent populations experience diabetes at an age, BMI, waist circumference, A1C, triglyceride levels, and fasting glucose concentrations which are often different than standard American Diabetes Association, World Health Organization and International Diabetes Federation screening thresholds (3, 7, 8, 10, 12, 13, 16, 47, 48). Furthermore, traditional risk factors such as BMI, waist circumference, A1C and triglycerides may not explain the high diabetes risk in low and middle-income countries (49). Therefore, prediction equations relying on these risk factors might underestimate diabetes risk. Studies conducted in Africa had relatively poor detection of diabetes, possibly because they used non-invasive risk criteria only. In addition, these equations were originally developed in European or Middle Eastern populations who may have different cardiometabolic risk profiles from African-descent populations (36, 38–40).

Varying definitions of diabetes diagnosis among prediction equation studies may have affected the performance of prediction equations. In African descent populations the use of A1C ≥6.5% (50), FPG ≥126 mg/dL (6) or self-reported diabetes diagnosis (51) to classify diabetes cases may lead to an underestimation in the absence of an OGTT.

Furthermore, we did not identify any prediction equations tested among African immigrants living in the diaspora. Despite the significant African immigrant populations in the United States and Europe and high rates of diabetes in these groups (7–9, 52, 53), no evidence has yet documented how well diabetes prediction equations detect diabetes in these groups in the diaspora. Two prediction equations identified in this review examined detection of diabetes among black Caribbean populations in Europe (Equations 2 and 9), but none examined detection among black Caribbean populations living in the Caribbean. In contrast, all seven prediction equations which were tested or re-estimated in the United States performed well among African Americans with AROC or C-statistic between 0.7 and 0.82.

These findings provide evidence that diabetes prediction equations which include invasive variables may show better discrimination of diabetes in populations of African descent than equations which rely on non-invasive variables alone (54). For the detection of diabetes, future studies in sub-Saharan Africa should consider the practicality of biochemical and hematological variables for improved discrimination.

## Limitations of this Review

Although the search attempted to be comprehensive, important articles published in peer reviewed journals not indexed in the PubMed, Scopus, and Embase databases may have been missed. To increase the likelihood of identifying articles published about specific communities in Africa and the diaspora we included names and adjectives of African countries. The use of racial terminology may also be a limitation at a global level. People of African descent who would self-identify as black in the US may not have the option to identify as such in other regions of the world. The heterogeneity within the African ancestry racial category is vast and presents a limitation of the findings in this review. However, detailed analysis by subgroups was limited.

It is also important to acknowledge the limitation of assuming that sub-Saharan African ancestry means having homogenous genetic, social and health profiles. Future studies should consider intra-ethnic variation among African-descent populations, and not lose sight of the importance of developing effective region- or ethnicity-specific prediction equations for better and earlier detection of diabetes.

## Conclusions

Diabetes is an enormous challenge in African descent populations. The absence of African-specific screening criteria contributes to significant underdiagnosis and underestimation of diabetes risk. To address this gap, the development and validation of diabetes prediction equations in African descent populations is urgently needed. Equations which use a combination of non-invasive and invasive variables are superior in the prediction of diabetes in African descent populations than equations that rely on non-invasive variables alone.

## Author Contributions

RM, AS, and MH-R conceived this research, conducted the literature review, wrote, and edited the manuscript. JA participated in the literature review, collection, and interpretation of the data. SB and TH participated in the revisions of the paper. All authors read and approved the final manuscript.

### Conflict of Interest

The authors declare that the research was conducted in the absence of any commercial or financial relationships that could be construed as a potential conflict of interest.
