# Supplementary material for: A Review of Diabetes Prediction Equations in African Descent Populations
Source: Front Endocrinol (Lausanne). 2019 Oct 1;10:663. doi: 10.3389/fendo.2019.00663 (PMC6779831; doi:10.3389/fendo.2019.00663)
Supplement: Supplementary file 1 [file Table_1.docx]

**Supplemental Material 1**

**Manuscript: A Review of Diabetes Prediction Equations in African Descent Populations**

**Authors:** Regine Mugeni, Jessica Y. Aduwo, Sara M. Briker, Thomas Hormenu, Anne E. Sumner, Margrethe F. Horlyck-Romanovsky.

**Literature Search, December 2018**

**PubMed**

**Search Terms:**

(predictive value of tests[majr] OR risk assessment[majr] OR predict[ti] OR predicted[ti] OR predicting[ti] OR predictive[ti] OR prediction[ti] OR predictions[ti] OR forecasting[majr] OR risk[ti] OR risks[ti] OR risk[majr] OR findrisc[tiab] OR framingham[tiab] OR aric[tiab] OR finnish[tiab] OR cambridge[tiab] OR leichester[tiab]) AND (equation[ti] OR equations[ti] OR model[ti] OR models[ti] OR score[ti] OR scores[ti] OR scoring[ti] OR algorithm[ti] OR algorithms[ti] OR algorithms[majr] OR findrisc[tiab] OR framingham[tiab] OR aric[tiab] OR finnish[tiab] OR cambridge[tiab] OR leichester[tiab]) AND (diabetes[ti] OR diabetic[ti] OR prediabetes[ti] OR prediabetic[ti] OR diabetes mellitus, type 2[majr]) AND (ethnic groups[majr] OR multiethnic[tiab] OR “multi-ethnic”[tiab] OR ethnicity[tiab] OR africa[tiab] OR african[tiab] OR africans[tiab] OR africa[mesh] OR afro[tiab] OR black[tiab] OR african continental ancestry group[mesh] OR african americans[mesh] OR Angola[tiab] OR Angolan[tiab] OR Benin[tiab] OR Beninese[tiab] OR Botswana[tiab] OR Motswana[tiab] OR Batswana[tiab] OR “Burkina Faso”[tiab] OR Burkinabe[tiab] OR Burundi[tiab] OR Burundian[tiab] OR Cameroon[tiab] OR Cameroonian[tiab] OR “Cape Verde”[tiab] OR “Cape Verdean”[tiab] OR “Central African Republic”[tiab] OR “Central African”[tiab] OR Chad[tiab] OR Chadian[tiab] OR Comoros[tiab] OR Comorian[tiab] OR “Republic of the Congo”[tiab] OR Congolese[tiab] OR Djibouti[tiab] OR Djiboutian[tiab] OR “Equatorial Guinea”[tiab] OR “Equatorial Guinean”[tiab] OR Equatoguinean[tiab] OR Eritrea[tiab] OR Eritrean[tiab] OR Ethiopia[tiab] OR Ethiopian[tiab] OR Gabon[tiab] OR Gabonese[tiab] OR Gambia[tiab] OR Gambian[tiab] OR Ghana[tiab] OR Ghanaian[tiab] OR Guinea[tiab] OR Guinean[tiab] OR “Guinea-Bissau”[tiab] OR “Bissau-Guinean”[tiab] OR “Ivory Coast”[tiab] OR Ivorian[tiab] OR Kenya[tiab] OR Kenyan[tiab] OR Lesotho[tiab] OR Mosotho[tiab] OR Basotho[tiab] OR Liberia[tiab] OR Liberian[tiab] OR Madagascar[tiab] OR Malagasy[tiab] OR Malawi[tiab] OR Malawian[tiab] OR Mali[tiab] OR Malian[tiab] OR Mauritania[tiab] OR Mauritanian[tiab] OR Mauritius[tiab] OR Mauritian[tiab] OR Mozambique[tiab] OR Mozambican[tiab] OR Namibia[tiab] OR Namibian[tiab] OR Niger[tiab] OR Nigerien[tiab] OR Nigeria[tiab] OR Nigerian[tiab] OR Rwanda[tiab] OR Rwandan[tiab] OR “Sao Tome and Principe”[tiab] OR “Sao Tomean”[tiab] OR Senegal[tiab] OR Senegalese[tiab] OR Seychelles[tiab] OR Seychellois[tiab] OR “Sierra Leone”[tiab] OR “Sierra Leonean”[tiab] OR Somalia[tiab] OR Somalian[tiab] OR “South Africa”[tiab] OR “South African”[tiab] OR “South Sudan”[tiab] OR “South Sudanese”[tiab] OR Sudan[tiab] OR Sudanese[tiab] OR Swaziland[tiab] OR Swazi[tiab] OR Tanzania[tiab] OR Tanzanian[tiab] OR Togo[tiab] OR Togoan[tiab] OR Uganda[tiab] OR Ugandan[tiab] OR Zambia[tiab] OR Zambian[tiab] OR Zimbabwe[tiab] OR Zimbabwean[tiab]) Filters: Publication date from 2000/01/01; English

**EMBASE**

**Search Terms:**

('predictive value'/exp/mj OR 'risk assessment'/exp/mj OR predict:ti OR predicted:ti OR predicting:ti OR predictive:ti OR prediction:ti OR predictions:ti OR 'prediction and forecasting'/exp/mj OR risk:ti OR risks:ti OR 'risk'/exp/mj OR findrisc:ti,ab OR framingham:ti,ab OR aric:ti,ab OR finnish:ti,ab OR cambridge:ti,ab OR leichester:ti,ab) AND (equation:ti OR equations:ti OR model:ti OR models:ti OR score:ti OR scores:ti OR scoring:ti OR algorithm:ti OR algorithms:ti OR 'algorithm'/exp/mj OR findrisc:ti,ab OR framingham:ti,ab OR aric:ti,ab OR finnish:ti,ab OR cambridge:ti,ab OR leichester:ti,ab) AND (diabetes:ti OR diabetic:ti OR prediabetes:ti OR prediabetic:ti OR 'non insulin dependent diabetes mellitus'/exp/mj) AND ('ethnic group'/exp/mj OR multiethnic:ti,ab OR 'multi-ethnic':ti,ab OR ethnicity:ti,ab OR africa:ti,ab OR african:ti,ab OR africans:ti,ab OR 'africa'/exp/mj OR afro:ti,ab OR black:ti,ab OR 'black person'/exp/mj OR 'african american'/exp/mj OR angola:ti,ab OR angolan:ti,ab OR benin:ti,ab OR beninese:ti,ab OR botswana:ti,ab OR motswana:ti,ab OR batswana:ti,ab OR 'burkina faso':ti,ab OR burkinabe:ti,ab OR burundi:ti,ab OR burundian:ti,ab OR cameroon:ti,ab OR cameroonian:ti,ab OR 'cape verde':ti,ab OR 'cape verdean':ti,ab OR 'central african republic':ti,ab OR 'central african':ti,ab OR chad:ti,ab OR chadian:ti,ab OR comoros:ti,ab OR comorian:ti,ab OR 'republic of the congo':ti,ab OR congolese:ti,ab OR djibouti:ti,ab OR djiboutian:ti,ab OR 'equatorial guinea':ti,ab OR 'equatorial guinean':ti,ab OR equatoguinean:ti,ab OR eritrea:ti,ab OR eritrean:ti,ab OR ethiopia:ti,ab OR ethiopian:ti,ab OR gabon:ti,ab OR gabonese:ti,ab OR gambia:ti,ab OR gambian:ti,ab OR ghana:ti,ab OR ghanaian:ti,ab OR guinea:ti,ab OR guinean:ti,ab OR 'guinea-bissau':ti,ab OR 'bissau-guinean':ti,ab OR 'ivory coast':ti,ab OR ivorian:ti,ab OR kenya:ti,ab OR kenyan:ti,ab OR lesotho:ti,ab OR mosotho:ti,ab OR basotho:ti,ab OR liberia:ti,ab OR liberian:ti,ab OR madagascar:ti,ab OR malagasy:ti,ab OR malawi:ti,ab OR malawian:ti,ab OR mali:ti,ab OR malian:ti,ab OR mauritania:ti,ab OR mauritanian:ti,ab OR mauritius:ti,ab OR mauritian:ti,ab OR mozambique:ti,ab OR mozambican:ti,ab OR namibia:ti,ab OR namibian:ti,ab OR niger:ti,ab OR nigerien:ti,ab OR nigeria:ti,ab OR nigerian:ti,ab OR rwanda:ti,ab OR rwandan:ti,ab OR 'sao tome and principe':ti,ab OR 'sao tomean':ti,ab OR senegal:ti,ab OR senegalese:ti,ab OR seychelles:ti,ab OR seychellois:ti,ab OR 'sierra leone':ti,ab OR 'sierra leonean':ti,ab OR somalia:ti,ab OR somalian:ti,ab OR 'south africa':ti,ab OR 'south african':ti,ab OR 'south sudan':ti,ab OR 'south sudanese':ti,ab OR sudan:ti,ab OR sudanese:ti,ab OR swaziland:ti,ab OR swazi:ti,ab OR tanzania:ti,ab OR tanzanian:ti,ab OR togo:ti,ab OR togoan:ti,ab OR uganda:ti,ab OR ugandan:ti,ab OR zambia:ti,ab OR zambian:ti,ab OR zimbabwe:ti,ab OR zimbabwean:ti,ab) AND [english]/lim AND [2000-2019]/py

**Scopus**

**Search Terms:**

( TITLE (predict  OR  predicted  OR  predicting  OR  predictive  OR  prediction  OR  predictions  OR  risk  OR  risks  OR  findrisc  OR  framingham  OR  aric  OR  finnish  OR  cambridge  OR  leichester )  AND  TITLE ( equation  OR  equations  OR  model  OR  models  OR  score  OR  scores  OR  scoring  OR  algorithm  OR  algorithms  OR  findrisc  OR  framingham  OR  aric  OR  finnish  OR  cambridge  OR  leichester )  AND  TITLE ( diabetes  OR  diabetic  OR  prediabetes  OR  prediabetic )  AND  TITLE-ABS-KEY ( multiethnic  OR  "multi-ethnic"  OR  ethnicity  OR  africa  OR  african  OR  africans  OR  africa  OR  afro  OR  black  OR  angola  OR  angolan  OR  benin  OR  beninese  OR  botswana  OR  motswana  OR  batswana  OR  "Burkina Faso"  OR  burkinabe  OR  burundi  OR  burundian  OR  cameroon  OR  cameroonian  OR  "Cape Verde"  OR  "Cape Verdean"  OR  "Central African Republic"  OR  "Central African"  OR  chad  OR  chadian  OR  comoros  OR  comorian  OR  "Republic of the Congo"  OR  congolese  OR  djibouti  OR  djiboutian  OR  "Equatorial Guinea"  OR  "Equatorial Guinean"  OR  equatoguinean  OR  eritrea  OR  eritrean  OR  ethiopia  OR  ethiopian  OR  gabon  OR  gabonese  OR  gambia  OR  gambian  OR  ghana  OR  ghanaian  OR  guinea  OR  guinean  OR  "Guinea-Bissau"  OR  "Bissau-Guinean"  OR  "Ivory Coast"  OR  ivorian  OR  kenya  OR  kenyan  OR  lesotho  OR  mosotho  OR  basotho  OR  liberia  OR  liberian  OR  madagascar  OR  malagasy  OR  malawi  OR  malawian  OR  mali  OR  malian  OR  mauritania  OR  mauritanian  OR  mauritius  OR  mauritian  OR  mozambique  OR  mozambican  OR  namibia  OR  namibian  OR  niger  OR  nigerien  OR  nigeria  OR  nigerian  OR  rwanda  OR  rwandan  OR  "Sao Tome and Principe"  OR  "Sao Tomean"  OR  senegal  OR  senegalese  OR  seychelles  OR  seychellois  OR  "Sierra Leone"  OR  "Sierra Leonean"  OR  somalia  OR  somalian  OR  "South Africa"  OR  "South African"  OR  "South Sudan"  OR  "South Sudanese"  OR  sudan  OR  sudanese  OR  swaziland  OR  swazi  OR  tanzania  OR  tanzanian  OR  togo  OR  togoan  OR  uganda  OR  ugandan  OR  zambia  OR  zambian  OR  zimbabwe  OR  zimbabwean ) )  AND  ( LIMIT-TO ( PUBYEAR ,  2018 )  OR  LIMIT-TO ( PUBYEAR ,  2017 )  OR  LIMIT-TO ( PUBYEAR ,  2016 )  OR  LIMIT-TO ( PUBYEAR ,  2015 )  OR  LIMIT-TO ( PUBYEAR ,  2014 )  OR  LIMIT-TO ( PUBYEAR ,  2013 )  OR  LIMIT-TO ( PUBYEAR ,  2012 )  OR  LIMIT-TO ( PUBYEAR ,  2011 )  OR  LIMIT-TO ( PUBYEAR ,  2010 )  OR  LIMIT-TO ( PUBYEAR ,  2009 )  OR  LIMIT-TO ( PUBYEAR ,  2008 )  OR  LIMIT-TO ( PUBYEAR ,  2007 )  OR  LIMIT-TO ( PUBYEAR ,  2006 )  OR  LIMIT-TO ( PUBYEAR ,  2005 ) OR  LIMIT-TO ( PUBYEAR ,  2004 )  OR  LIMIT-TO ( PUBYEAR ,  2003 )  OR  LIMIT-TO ( PUBYEAR ,  2002 )  OR  LIMIT-TO ( PUBYEAR ,  2001 ) OR  LIMIT-TO ( PUBYEAR ,  2000 ) )  AND  ( LIMIT-TO ( LANGUAGE ,  "English" ) )
